# Guided Imagery for Stress and Symptom Management in Pregnant African American Women

**DOI:** 10.1155/2014/840923

**Published:** 2014-02-25

**Authors:** Nancy Jallo, R. Jeanne Ruiz, R. K. Elswick, Elise French

**Affiliations:** ^1^Virginia Commonwealth University, School of Nursing, P.O. Box 980567, Richmond, VA 23298, USA; ^2^Texas Tech University, Gayle Greve Hunt School of Nursing, 415 East Yandell, El Paso, TX 79902, USA; ^3^Riverside Medical Group, 856 J Clyde Morris, Newport News, VA 23602, USA

## Abstract

The purpose of this study was to evaluate the efficacy of a guided imagery (GI) intervention for stress reduction in pregnant African American women beginning early in the second trimester. This prospective longitudinal study of 72 women used a randomized controlled experimental design with two groups conducted over 12 weeks. The intervention was a CD with 4 professionally recorded tracts designed and sequenced to influence study variables. Participants in both GI and usual care (UC) completed measures and donated 5 cc of blood at baseline, 8 weeks and 12 weeks. Participants also completed a daily stress scale. A mixed-effects linear model tested for differences between groups for self-reported measures of stress, anxiety, and fatigue as well as corticotrophin releasing hormone (CRH), a biologic marker of stress. Significant differences in perceived stress daily scores and at week 8 but not week 12 were found in the GI group compared to UC group. The GI group reported significantly less fatigue and anxiety than the UC group at week 8 but not week 12. There were no significant differences in CRH levels between groups. Results suggest that GI intervention may be effective in reducing perceived stress, anxiety, and fatigue measures among pregnant African American women.

## 1. Introduction

Maternal stress has been associated with pregnancy complications such as hypertension and preeclampsia as well as negative perinatal outcomes such as intrauterine growth restricted (IUGR), low birth weight (LBW) infants, preterm birth (PTB), and neuropsychological developmental delays of affected offspring [1–3]. Perceived stress is often associated with symptoms such as anxiety and fatigue [2, 3] and these related symptoms may increase the deleterious effect of stress on health and birth outcomes. For example, state and trait anxiety during pregnancy has been associated with stress and has been found to significantly predict gestational age and preterm birth [4, 5]. Likewise, maternal fatigue has been found to be positively associated with stress [6] and anxiety [7, 8] and is recognized as a symptom of these mental states [9]. There is an association between fatigue and antenatal morbidity [10] and adverse perinatal outcomes such as PTB [7] and risk of preterm premature rupture of membranes in nulliparous women [11].

Of equal importance is the major disparity in rates of stress and pregnancy complications between African American women and Caucasian or Hispanic women [12]. Research has demonstrated that AA women experience significantly more prenatal stress [13, 14] and it has been proposed that they have increased susceptibility to the negative effects of psychosocial stressors when compared to Hispanic or Caucasian women [12]. There is also the possibility of health disparities related to fatigue. For example, of women with preterm labor, African Americans had higher level of fatigue than Caucasians [15]. Despite the high prevalence and potential negative perinatal consequences, very few studies have focused on interventions to decrease prenatal stress and the related symptoms of anxiety and fatigue in pregnant African American women.

The pathway from maternal stress to negative health outcomes is a multifaceted and complex process which represents the connection between the mind and the body [16]. The perception of stress in the mind triggers biological changes in the body leading to physiologic reactions that can have detrimental effects on health outcomes [17]. This connection is mediated by the hypothalamic-pituitary-adrenal (HPA) pathway with corticotrophin releasing hormone (CRH) playing a major role in the physiologic response to stress [17]. Studies have demonstrated that stress and anxiety during pregnancy are associated with elevated levels of CRH [2]. Evidence from studies suggest that women with significantly elevated levels and/or significantly accelerated rate of CRH increase over the course of pregnancy have an increased risk for pregnancy complications such as preterm birth [2, 18], intrauterine growth restriction [19], and reduced birth weight [20]. In addition, in the general population, CRH and neuroendocrine hormones mediated by CRH have been associated with fatigue [21] and anxiety [22].

Given the mind-body connection between maternal stress and adverse health events, it is possible that a complementary and alternative medicine (CAM) intervention may be effective in reducing self-reported and biologic measures of stress during pregnancy [23]. However, many of the CAM interventions are presented in groups or one-on-one, which requires participant's scheduled time, child care, transportation, health-care provider time, and cost—all of which present potential barriers to participation and implementation into clinical practice. Guided imagery (GI) is a powerful, mind-body CAM therapy that is an economic, simple, and easy to use intervention that can be easily delivered in a self-help format thus eliminating potential barriers inherent in other CAM interventions [24]. Given these potential benefits and today's demands on the health care system, the authors selected GI for further investigation in this study.

GI is a psychophysiological dynamic modality in which a person imagines and experiences an internal reality in the absence of external stimuli [25]. The mechanism of action may be related to the power of GI to send messages and information from the brain to the central nervous system and thus connect with physiological processes [26]. GI represents a basic principle of psychophysiology in that every thought has a physiologic response. When a mental image is experienced, there is an associated emotion connecting the feeling state with the mind and body leading to a physiologic change [25, 27]. Thus, a GI intervention may decrease perceived stress and associated symptoms which, in turn, may provide a positive influence on neuroendocrine factors, such as CRH.

The use of GI has been reported to reduce self-reported measures of stress, anxiety, and fatigue as well as neuroendocrine measures of stress such as cortisol among nonpregnant participants [27–30]. However, there is limited research investigating this intervention on self-reported and biologic measures of stress and associated symptoms among pregnant women in a prenatal care setting. A longitudinal pilot study examining a GI intervention in pregnant African American women reported a significant decrease in state anxiety and weekly numeric rating scale of stress (NRSS) scores over time compared to the usual care (UC) group but not perceived stress scores or level of CRH over time [31].

Hospitalized pregnant women with preterm labor were found to benefit from a GI intervention. In Taiwan, this population was found to have lower state anxiety scores but not perceived stress scores compared to control group immediately after 3 sessions and over time until delivery [32]. Women hospitalized with preterm labor in the US reported significantly lower mean perceived stress levels after listening to a GI intervention compared to preintervention stress levels [33]. However, background music was added to the GI, thus making it difficult to isolate the effect of imagery.

Not all studies have reported a reduction in anxiety. For example, the use of imagery during prenatal classes reported no significant differences in anxiety state [34]. Likewise, a brief GI intervention did not significantly reduce state anxiety in healthy pregnant women [35]. Because both of these studies investigated a single 10-minute session of GI, the short exposure to the intervention rather than the intervention itself may have contributed to the lack of findings.

Because imagery links perceptions, emotions, and physiological responses, the selection of appropriate images is of utmost importance [36]. Previous research has investigated imagery exercises in pregnant women for general stress management [31, 32, 35] or childbirth [34] but no studies reported including imagery exercises for pregnancy-specific stress, which is inherent during pregnancy [4]. The effect of the addition of healthy pregnancy-related images to the previously studied general stress management images on maternal stress and associated symptoms is unknown and warrants further investigation.

While the results of these studies are promising, variations in research design, dosage of intervention, and selected study variables limit the applicability of these findings. Additional research addressing these limitations is warranted. Therefore, the purpose of this study was to investigate the effects of a 12-week professionally developed GI intervention on self-reported and biologic measures of perceived stress and the associated symptoms of anxiety and fatigue in pregnant African American women.

## 2. Materials and Methods

### 2.1. Study Design

This study used a repeated measures, two-group randomized experimental design to examine the effect of GI on perceived stress, and the associated symptoms of anxiety and fatigue, as well as CRH, a neuroendocrine stress measure.

### 2.2. Participants

Participants were recruited from 2 academic obstetric clinics affiliated with the Virginia Commonwealth University Health System and the Riverside Regional Health System. Inclusion criteria included being (1) pregnant African American women between 14 and 17 weeks gestation, (2) ≥18 years of age or older, (3) able to read, write, and understand English, and (4) able to verbalize a source of social support. Exclusion criteria included (1) multiple pregnancy, (2) cervical cerclage, (3) current use of oral corticosteroids, (4) uterine or cervical abnormality, (5) dissociative disorders, borderline personalities, and psychotic pathology, (6) medical and/or pregnancy complications, and (7) current use of GI techniques. Criteria were selected to reduce sources of variability between and within subjects to include (1) variables that could potentially influence CRH levels such as ethnicity, development and growth of the placenta, gestational age of the pregnancy, use of glucocorticoids, and medical and/or pregnancy complications potentially associated with infection/inflammation [1] and (2) variables that could potentially influence participant safety or treatment effects such as use of unsupervised guided imagery in individuals with disruption in the functions of consciousness, memory, or identity as well as social support, which has been reported to be a buffer of the stress response [16]. Criteria were initially measured when participants completed the Demographic and Health History form and the Health History component was reviewed for changes at every study visit.

**Figure 1:**
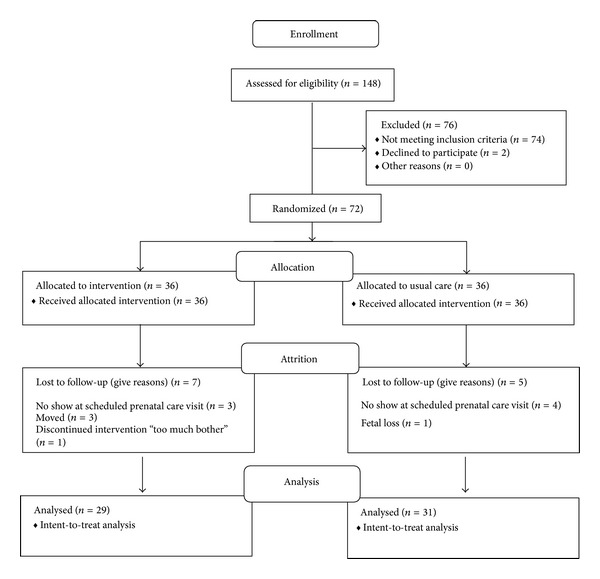
Flow diagram of enrollment and randomization.

A total of 148 potential participants were assessed for eligibility. Of these 148 women, 74 did not meet inclusion criteria, 2 were eligible but declined to participate, and 72 pregnant African American women between 14 and 17 weeks gestation were enrolled after completing the informed consent process (Figure 1).

### 2.3. Intervention

The 12-week intervention consisted of a CD with 4 GI tracts, each 20 minutes in length. The script for each CD was developed and professionally recorded by the author certified in GI. Key components included relaxation, focused breathing, and a variety of multisensory images to promote reduction of stress and anxiety as well as to restore levels of energy. The intervention contained multiple styles of imagery to include (1) feeling state imagery designed to shift the participant's mood to one of peace and calm, (2) end state imagery suggesting that participants see themselves the way they wish to be, and (3) energetic imagery focusing on restoring normal levels of energy [37].

The content and order of the CDs were sequenced to focus on the desired outcomes and were based on examples in the professional literature and the Academy for Guided Imagery [31, 36, 38–41]. CD tract number 1 was designed to promote familiarity with relaxation and imagery. Instructions were provided for CD use, focused breathing, and relaxation techniques. Participants were encouraged to imagine themselves in a peaceful, serene, safe, and secure personal place to rest and let go of their anxiety, worries, or concerns and emerge from their imagery with a sense of feeling refreshed and recharged. This feeling state image of a pleasant safe scene focused on feelings of peace, calm, and relaxation. CD tract number 2 included a shortened version of the content in CD tract number 1 and added feeling, end-state, and energetic images associated with decreases in stress and stress-related symptoms, decreases in physiological and psychological arousal, and energy restoration [37, 40, 42]. For example, participants were encouraged to “imagine a large ball of energy” and visualize the image's form, color, flow, and effects on how they are feeling. Language cues included suggestions for the desired physiological or psychological change (e.g., *“I am calm, I am relaxed, I am energized, I am invigorated with a powerful sense of well-being*”). It is these images that potentially mediate the communication between perception, emotion, and physiologic change and may affect a physiologic process such as reducing the stress reaction and the related stress symptoms [26]. CD tract number 3 (week 3) included a shortened version of focused breathing and relaxation along with the addition of feeling state and energetic images associated with decreases in stress, anxiety, and fatigue as well as end-state imagery, whereby the participant was invited to imagine rehearsing and successfully meeting the stressful challenges in their daily life. The inclusion of such imagery was important as it can be useful to bring things into real life and assist the participants in meeting those challenges [40]. This tract also contained cues such as “*letting the feelings of calmness and relaxation carry over with you into a fully alert state*” to promote sustained effects of the GI. CD tract number 4 (week 4) included a shortened version of focused breathing and relaxation along with feeling, end-state, and energetic images associated with decrease in stress, anxiety, and fatigue. Because pregnancy-related worries have been identified as major stressors and sources of anxiety in African American women [43], feeling and end-state images related to a healthy pregnancy and baby such as “*Imagine your baby tucked away safe inside your body—safe and cozy—cushioned and protected*” combined with positive affirmations such as “*More and more I can let go of worrying about things and focus on my own inner peace and stillness*” were also included to target potential pregnancy-specific stressors.

### 2.4. GI Group

The R-GI group continued usual care with the addition of the GI intervention. Participants were instructed to listen to the CD once a day for 12 weeks in a recommended order for weeks 1–4 and used in any order for weeks 5–12. This plan was based on a study reporting that some women got tired of hearing the same script after three weeks [44] as well as feedback from a previous study [45]. The design called for daily practice for twelve weeks because (1) daily practice is an important component of skill acquisition [39], (2) it has been demonstrated that stress-related symptoms as well as the individual's appraisal of stress improves within 8–12 weeks of relaxation-based programs [46], and (3) GI intervention must be introduced early enough and last long enough to decrease stress and potentially impact levels of CRH.

### 2.5. UC Group

Participants in the UC group continued their usual obstetric care. To reduce the possible effect of researcher attention, a member of the researcher team called participants every week to ask if they have completed the NRSS measures for that week.

### 2.6. Measures

#### 2.6.1. Demographic and Health History

A self-reported demographic and health history questionnaire was developed for this study to assess participants' basic demographic information as well as medical, mental, and/or obstetric risk factors and current health behaviors. Estimated gestational age (EGA) was determined by the first day of the last menstrual period and, if possible, confirmed by ultrasound [47].

#### 2.6.2. Perceived Stress Scale (PSS)

This is a 10-item, self-reported measure of global perceived stress that measures the degree to which a respondent appraises one's life as being stressful during the past month [48]. The score range is 0 to 56 with a higher score indicating a higher level of perceived stress. The PSS has accrued considerable reliability and validity data with internal consistency alphas at 0.85 and reliability at 0.87 and is widely used in the general population and in studies of pregnant women [2, 49, 50]. Cronbach's alpha for this study was 0.87.

#### 2.6.3. Numeric Rating Scale of Stress (NRSS)

This is a unidimensional scale developed for the purpose of quantifying the intensity of stress [31, 51]. The NRSS measures the participant's level of stress on a continuum of intensity using an 11-point scale, with 0 representing *no stress *and 10 representing* the worse stress imaginable.* A numeric rating scale is considered to be a simple yet sensitive measure of subjective phenomenon and has been used in anxiety, relaxation, and GI research in the general and pregnant population [31, 36, 52, 53].

#### 2.6.4. State-Trait Anxiety Inventory (STAI)

This is a self-report measure consisting of two 20-item subscales measuring state anxiety and trait anxiety [54]. The state anxiety responses range from *not at all* (1) to *very much so* (4), whereas the trait anxiety responses range from 1 (*almost never*) to 4 (*almost always*). Scores for each subscale range from 20 to 80 with a higher score indicating a higher level of state or trait anxiety. The STAI is considered a reliable measure with Cronbach's alpha reliability reported between 0.86 and 0.93 [54] and has been used in the stress-related research and pregnant women [2, 31, 55]. Cronbach's alpha for this study was 0.89.

#### 2.6.5. Brief Fatigue Inventory (BFI)

This is a 9-item self-report measure that assesses the severity of fatigue and interference on daily functioning over the past 24 hours [56]. Three items address fatigue severity (worst and usual fatigue during the past 24 hours and current fatigue) and 6 items address interference on an 11-point numeric rating scale. The BFI is considered a reliable measure with Cronbach's alpha reliability ranging from 0.82 to 0.97 in clinical trials [56] and has been used in stress-related and intervention research [29, 57]. Cronbach's alpha for this study was 0.87.

#### 2.6.6. Daily Practice Log

Frequency of GI CD tract use, barriers to use, and perceived benefits of the intervention were assessed by participants' documentation on a daily practice log developed for this study.

#### 2.6.7. Corticotrophin Releasing Hormone (CRH) Assay

Venous blood samples were drawn from a subset of participants using standard venipuncture procedures in the clinic during a prenatal care visit. Blood samples were collected into tubes containing the anticoagulant ethylenediaminetetra-acetic acid (EDTA) and the protease inhibitor aprotinin (0.6 TIU/mL of blood) to prevent degradation of the sample [58]. The samples were placed on ice and transported to the Virginia Commonwealth University School of Nursing P30 Center of Excellence Measurement Core laboratory, where the samples were centrifuged at 1,6000 ×g for 15 minutes at 4° Celsius and immediately stored at −70°C. Once all samples were obtained from the enrolled participants, the frozen plasma samples were shipped overnight on dry ice in an approved biohazard container to the University of Texas at Austin School of Nursing biobehavioral laboratory for methanol extraction and CRH radioimmunoassay per an established and published protocol [58].

### 2.7. Procedure

#### 2.7.1. Baseline (Time 1)

Eligible women who consented and agreed to participate were randomized using a computer generated masked assignment list using envelopes to the GI or UC group. At time 1, participants completed the demographic and health history questionnaire and the PSS, STAI, and BFI. Blood was drawn for plasma CRH. Folders containing the daily logs, instructions for completion of the NRSS, and contact information were given to all participants. Participants in the GI group received the CD, CD player, and extra batteries and were individually introduced to GI by a member of the research team.

#### 2.7.2. Time 2

Approximately 8 weeks (EGA 22–25 weeks) from the initial visit, a member of the research team met with the participants at the prenatal visit. Participants completed the PSS, STAI, and BFI and had blood drawn for plasma CRH. The logs from the previous 8 weeks were collected.

#### 2.7.3. Time 3

Approximately 4 weeks from time 2 (EGA 26–29 weeks), participants completed the PSS, STAI, and BFI and had blood drawn for plasma CRH. The logs from the previous 4 weeks were collected.

#### 2.7.4. Participant Procedures at Home for Weeks 1–12

The NRSS was completed daily. The UC group was instructed to complete it in the evening and the GI group before and after listening to the CD. In addition, the GI participants were asked to record any perceived benefits or barriers to using the CD on the daily practice log. The research team telephoned all participants weekly to inquire about their wellbeing and any difficulties in completing the NRSS forms.

### 2.8. Data Analysis

Descriptive statistics were used to summarize demographic characteristics. At baseline (Time 1), groups were compared using two-sample *t*-tests for continuous variables and chi-square tests for categorical variables.

A mixed-effects linear model was used to test for differences between the GI intervention group and the UC group across time for the behavioral variables (PSS, STAI, and BFI) and biologic variable (CRH). Following the intent to treat principle, all participants with postbaseline data were used in the mixed effects linear model. The fixed effects included visit number (baseline (visit 1), visit 2, and visit 3), intervention group (GI and UC), and visit by intervention group interaction. Participant was modeled as a random effect. CRH was positively skewed; thus, a log-transformation served the dual role of normalizing the data and stabilizing the variance.

NRSS scores were obtained daily from each participant. In the GI group, two NRSS sores were obtained (prior to and after listening to the GI intervention) and one NRSS score in UC group was obtained. These daily data were averaged to obtain weekly means (weeks 1–12). Thus, each subject had as many as 12 weekly averages. Two different questions are posed regarding these data. The first is “do the weekly means differ from before to after intervention for the subjects within the GI group?” The mixed-effects linear model used to test this question contained fixed effects for week (1–12), time (pre- or post-CD), and a week by time interaction and a random effect for participant. In order to model the doubly repeated measures (pre/post and across 12 weeks), an unstructured variance structure was used to model the repeated time (pre/post) and a compound symmetric variance structure was used to model the repeated week (1–12). The second question asked of the daily data was “do the post-GI group means differ from the UC group means across the weeks?” The data used to answer this question was the post-GI data and the UC group data. The mixed-effect linear model used to test this question contained fixed effects for week (1–12), intervention group (GI versus UC), and a week by intervention group interaction and a random effect for participant.

Because this was a pilot study, no adjustment for multiplicity was used; the results from this trial should be regarded as exploratory and should be confirmed in a larger trial. JMP and SAS were used for all data analyses.

### 2.9. Ethical Approval

The study protocol was approved by the Institutional Review Boards (IRB) of Virginia Commonwealth University and Riverside Regional Medical Center.

## 3. Results 

### 3.1. Demographics

Participants ages ranged from 18 to 39 years with a mean age of 24.26 years (1.06) (Table 1). The mean estimated gestational age (EGA) at enrollment was 15.43 (0.16) weeks and 21 participants (29%) were primigravida and for 23 participants (32%) this was their second pregnancy. The majority of participants were not married (86%, 62/72), were educated after high school (51%, 37/72), reported that they were not employed out of the home (60%, 43/72), and had an income of <$15,000 (68%, 49/72). Thirteen participants (18%, 13/72) reported smoking and 6 (8%, 6/72) reported current use of alcohol. The majority (85%, 60/71) stated they did not use any stress management techniques. There were no significant differences between groups on demographic variables or baseline PSS and STAI scores and CRH concentrations between the groups (Table 1). The groups were similar with respect to smoking and alcohol use and utilization of stress management techniques. Of the 72 women who were randomized and completed the baseline measures, 12 (12/72) did not complete the 12-week study. Reasons included the following: (1) seven did not attend the scheduled prenatal care visit and were lost to follow up; (2) three women moved; (3) one experienced a fetal loss; and (4) one GI participant withdrew because it was “too much bother” (Figure 1).

### 3.2. Perceptions of Stress

**Figure 2:**
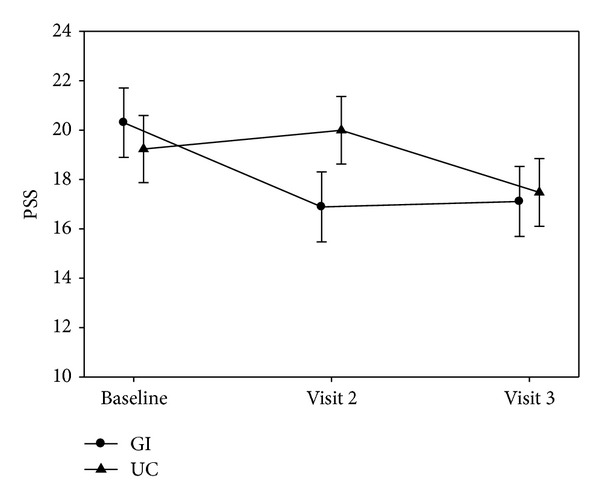
Differences in perceived stress scores over time between the GI and UC group.

The mean PSS scores for the GI and UC groups for each time point are presented in Table 2. While the UC group's perceived stress score mean increased slightly from 19.23 to 19.99 from baseline to week 8, the GI group's perceived stress score mean decreased from 20.30 to 16.89, representing a statistically significant reduction for the GI group. At week 12, there was no statistical difference between the groups. These results are illustrated in Figure 2.

**Figure 3:**
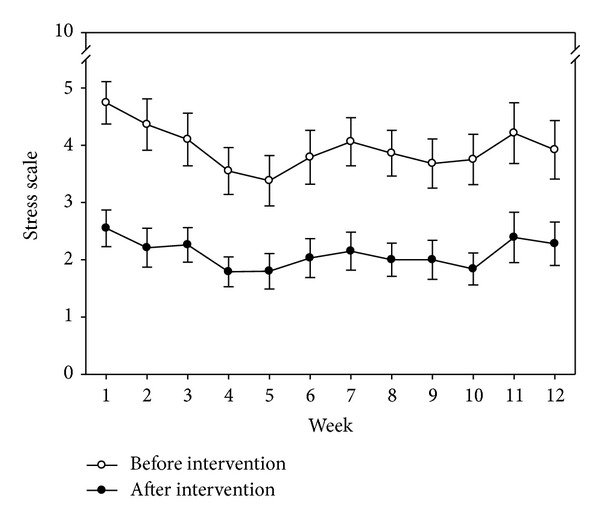
Pre- and postintervention numeric rating scale of stress scores over 12 weeks in the GI group.

**Figure 4:**
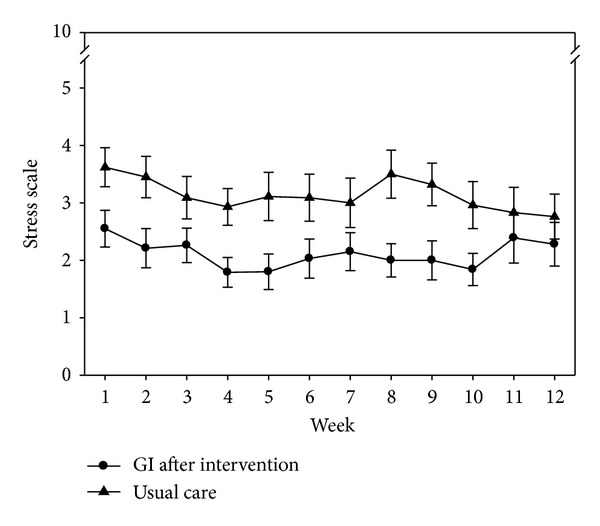
Differences in UC and GI postintervention numeric rating scale of stress scores over 12 weeks.

The first question of the NRSS variable was “do the weekly means differ from before to after intervention for the subjects within the GI group?” From the mixed model analysis, time (pre- versus post-CD) was statistically significant (*P* < 0.001). The week (1–12) (*P* = 0.081) and week by time interaction (*P* = 0.457) were not significant.  Figure 3 contains the plot of the least square means and illustrates the approximate 2-point reduction in NRSS from before to after intervention. The second question asked of the NRSS variable was “do the post-GI means differ from the UC group means?” From the mixed model analysis, group differences (GI versus UC) was statistically significant (*P* < 0.019). The week (1–12) (*P* = 0.255) and week by time interaction (*P* = 0.376) were not significant.  Figure 4 contains the plot of the least square means and illustrates the reduction in NRSS scores between the GI and UC group.

### 3.3. Associated Symptoms

#### 3.3.1. Anxiety

The mean STAI scores for the GI and UC groups for each time point are presented in Table 2. While state anxiety scores decreased in the GI group at time 2 as compared to UC, there was not a significant difference between the 2 groups. There was a significant reduction in trait anxiety scores in the GI group at 8 weeks (time 2) compared to the UC group. There were no significant differences in state or trait scores between the groups at 12 weeks (time 3).

#### 3.3.2. Fatigue

**Figure 5:**
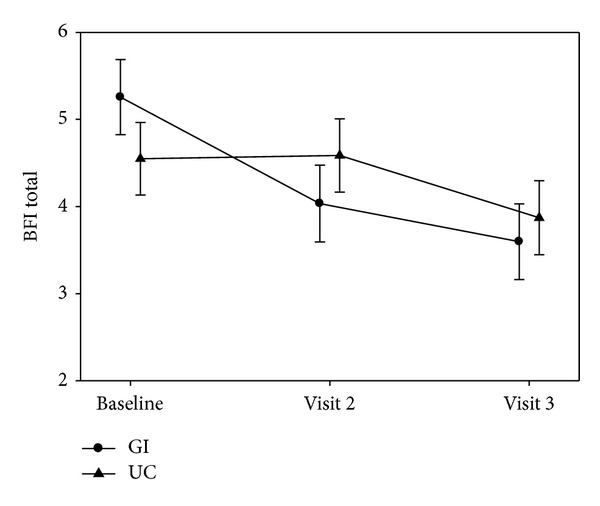
Differences in Brief Fatigue Inventory Scores over time between the GI and UC group.

The mean BFI scores for the GI and UC groups for each time point are presented in Table 2. There was a significant reduction in BFI scores in the GI group compared to the UC group at 8 weeks (time 2) but not 12 weeks (time 3).  Figure 5 illustrates this reduction in scores.

### 3.4. CRH

Due to laboratory assay problems beyond our control, the plasma of only 31 participants (GI = 16; UC = 15) were used for the CRH analysis. The median (pg/mL) and range for CRH levels are presented in Table 2. All participants who were included in the data analysis had a sequential rise of CRH levels over time. There were no statistically significant differences in CRH levels between the groups at 8 or 12 weeks.

### 3.5. Daily Practice Log

The frequencies of CD tract usage from week 5–12 are presented in Table 3. The weekly frequency data represents the number of times the GI group participants listened to the CD tract during the 7 days of the specified week as documented in the daily practice log. During week 5–12, participants were instructed to select and use the CD tracts in any order. During this time, participants listened to all tracts. Participants reported the highest frequency of CD tract usage as follows: CD number 1 during weeks 5, 6, 9, and 19; CD number 2 during week 7; CD tract number 3 during weeks 8 and 11; and CD tract number 4 during week 12. The adherence rate for listening to the GI CD tracts during weeks 5–12 ranged from 75.86% to 90.15%.

One hundred percent of GI group participants wrote in their daily logs at least 1 benefit they had received by listening to the intervention and most participants documented multiple benefits. Comments such as “definitely helped me calm down and not think of anything negative,” “I felt like a weight was lifted,” “it helped me deal with stress better,” “I let the energy come so I could be stress-free,” “ it brings me closer to my baby,” “ease feelings I was having about the baby,” “tiredness and headache went away,” and “learned how to control myself when I am in a tight spot” are just several of many comments describing the perceived benefit of the intervention.

The majority of participants reported no perceived barriers. Of those documented, all barriers pertained to the delivery system (e.g., CD player skipped and batteries ran low) or environmental disruptions that made it difficult to concentrate.

## 4. Discussion

### 4.1. Study Outcomes

The purpose of this study was to investigate the effects of GI on self-reported and biologic measures of stress and the associated symptoms anxiety and fatigue in pregnant African American women. There was a statistically significant reduction in perceived stress scores at 8 weeks (time 2) but not at 12 weeks (time 3) in the GI compared to UC group. The significant decrease in PSS scores for the GI group at 8 weeks has not been reported in previous studies with pregnant women [31, 32]. A possible explanation for this finding may be related to the selection of images for this study. While a previous study included images related to focused breathing, relaxation, stress, and stress-related symptoms, as well as mental rehearsal imagery [31], this study included those images but added a 4th CD tract with images related to a healthy pregnancy and baby. Pregnancy is a complex and dynamic condition wherein the pregnant woman and fetus have an intricate relationship [59]. Thus, it would be no surprise that the health of the pregnancy and baby is of concern to the pregnant woman. It is possible that the inclusion of affirmations such as “*I know my baby is tucked away inside my body—safe and cozy—cushioned and protected* and images of *how secure and comfortable and safe the baby is in your body every cell in your body supporting this wonderful baby*” promoted a sense of maternal wellbeing and a reduction in perceived stress. While there was a significant difference in PSS scores at 8 weeks (time 2), this was not the case at 12 weeks (time 3). This did not appear to be due to an increase in PSS mean scores in the GI group but rather a decrease in the UC group mean PSS scores, while the GI group mean remained constant. However, even though the GI participants were able to maintain their initial changes at 12 weeks, they did not demonstrate further reduction in PSS scores.

The NRSS weekly mean stress scores were significantly lower in the GI compared to UC group and, in the GI group, the NRSS scores were significantly lower after intervention compared to before intervention. These findings are consistent with prior reported research [31] and add evidence to the effectiveness of this intervention to reduce perceived stress over the course of a single week. This is important because daily stressors affect wellbeing and can accumulate over time [60]. Our body responds to daily events and attempts to maintain homeostasis by an active process often referred to as allostasis [60]. It is the increase of the daily stress events and/or the inefficient management of allostasis that results in an increase in allostatic load and potentially negative health outcomes [17]. The results suggest that GI had an immediate effect on decreasing day-to-day perceived stress, which potentially may impact allostatic load and ultimately health outcomes.

While the GI group's state anxiety scores trended downward and the UC group's state anxiety scores remained fairly constant at 8 weeks, there were no significant differences in the state anxiety scores at time 2 or 3. While previous studies [31, 32] reported a reduction in state anxiety scores following a GI intervention, the findings of this study are consistent with those studies [34, 35] which found no reduction in state anxiety scores. While state anxiety is a common emotional reaction to stress and has been associated with negative health outcomes, recent research suggests that pregnancy anxiety, a newer concept, is one of the most potent maternal risk factors for adverse outcomes [4]. While closely associated with state anxiety, pregnancy anxiety is a particular emotional state closely related to state anxiety but more contextually based to concerns about the current pregnancy [4]. Thus, it is possible that a general measure of state anxiety would not capture the multidimensional aspect of anxiety during pregnancy. If so, it is conceivable that an intervention designed to reduce anxiety during pregnancy may not affect a general measure of anxiety.

There was a significant difference in trait anxiety scores with the GI group reporting decrease levels at time 8 but not time 12 compared to UC group. Prior studies [31, 32, 34, 35] have reported on effect of GI on state anxiety but not trait anxiety. The significant reduction in trait anxiety reported in this study is an important finding as trait anxiety levels during pregnancy have been associated with negative neonatal development and behavior. For example, increased levels of trait anxiety during pregnancy have been shown to adversely influence fetal hemodynamics [61] and are associated with lower infant orientation and self-regulation scores [62], reports of difficult infant temperament [63], and slower growth of the infant's hippocampus over the first 6 months of life [64]. Thus, an intervention that decreases maternal trait anxiety may have long-term implications for fetal and infant development as well as maternal-infant interactions.

Fatigue at 8 weeks (time 2) was decreased in the GI group compared to UC group. We believe this is the first published study to document the effects of GI on fatigue in pregnant African American women. This finding is consistent with reports of researchers examining the effects of GI on fatigue in other populations [29, 65]. There are potentially several explanations for this finding. First, since the GI group reported lower perceived stress scores weekly and at 8 weeks and stress has been found to be associated with fatigue, it is reasonable that the GI group would experience a decrease in fatigue. Another possible explanation is related to sleep quality. One of the most common causes of fatigue during pregnancy is disturbed sleep [66]. Sleep disturbances during pregnancy may be the result of difficulty falling asleep and/or maintaining sleep [67] due to midsleep awakenings [68] and nightmares [69] which results in difficulty maintaining sleep [67]. In fact, frightening dreams or nightmares have been cited as a common cause of midsleep awakening by 72% of pregnant women [67]. The comments written by the GI participants in the daily logs regarding their perceived benefits of the intervention on their sleep may offer an explanation for the effect of GI on fatigue. Comments such as “they helped me to fall asleep,” “stay asleep,” “improved my dreams,” and “helped me sleep longer” suggested that the intervention potentially improved the participants' sleep and, thus, decreased fatigue. As total time spent asleep has been reported to play a significant role in perceived fatigue[68], this is a plausible explanation.

As expected, the CRH increased at each measurement. In a normal pregnancy, levels of CRH in maternal blood increase exponentially from weeks 15 to 36 gestation with a significant rise at weeks 26 to 30 [49]. It is the significantly elevated and/or accelerated rate of CRH increase over the course of the pregnancy that is associated with negative outcomes [49]. Given the small sample size analyzed and the expected wide ranges of CRH during pregnancy, it is not surprising that no significant differences were found.

The intervention was well received by the GI group. The GI participants had never used GI prior to this study and were open to participating and listening to the intervention. As evidenced by the written comments in the daily practice log, the participants found the intervention helpful in feeling less stressed and calmer, relieving symptoms, and soothed pregnancy-related concerns. The positive comments coupled with the fact that only one participant withdrew because the intervention was “too much bother” suggest that CAM interventions are potentially acceptable to African American pregnant women. This is an important finding given the current health disparity in the use of CAM interventions in the United States. Existing data suggest that CAM use is lower among African Americans compared to non-Hispanic Whites and Asian Americans [70]. In this study, AA women participated at a high level and used a variety of GI CD tracts over the course of 12 weeks. The results of this study highlight the importance of health care providers discussing CAM interventions and introducing effective yet inexpensive therapies such as GI particularly to pregnant African American women.

### 4.2. Research Implications

There are multiple research implications resulting from this study. An interesting and important area for a future focus is to examine if a booster (s) between 8 and 12 weeks would continue to help improve the stress levels of the group receiving GI as boosters have been found to sustain and/or improve the effectiveness of health behavior interventions [71]. A larger randomized controlled trial for efficacy in relationship to improvement in infant outcomes is also needed. Future research might also consider testing an allostatic load score using a combination of stress and anxiety with biological markers to predict preterm birth and test if this allostatic load is then changed as a composite score. Additional studies testing the effect of GI in pregnancy on anxiety might also benefit from including pregnancy-related anxiety as well as generalized anxiety measures. An interesting and potentially important finding out of this study is the effect of GI on fatigue. Because sleep quality is a predictor of maternal mood, stress, and fatigue [72], future work might include a sensitive measure of sleep quality in relationship to the GI use. Finally, a larger randomized controlled trial examining the effect of the GI use on biological markers such as CRH is also warranted.

### 4.3. Study Limitations

Our results must be balanced with study limitations. In terms of adherence to the intervention, the majority of participants reported daily use of the GI intervention. However, self-reported measures are dependent on the participant's accurate reporting. It is possible that participants under- or overreported information or did not complete the form in a timely manner, which are all potential threats to validity. Future studies could address this limitation through the use of expanding mobile technology that can facilitate self-report data collection and validate intervention use. In some instances, participants' health history information was self-reported and the recruitment site was unable to verify the information. This is a potential limitation when trying to control for confounding factors. For example, fatigue during pregnancy is known to be influenced by coexisting factors such as anemia, excessive weight gain, diet, and physical activity [8]. Thus, the differences in fatigue between groups could be confounded by other factors. In addition, the CRH sample that was analyzed was small which limited identifying treatment effects.

## 5. Conclusion

This study investigated the effects of a GI intervention on maternal stress and associated symptoms of anxiety and fatigue in pregnant African American women beginning in the second trimester. The findings demonstrated significant effects of the GI intervention on perceived stress, anxiety, and fatigue. Within the GI group, the intervention had an immediate effect as evidenced by significant differences in pre- and postintervention averaged daily scores and the GI group reported significantly lower perceived stress scores compared to UC group at 8 weeks. In addition, the GI group reported significantly less trait anxiety and fatigue compared to UC group at 8 weeks.

This study also provides direction for consideration in future studies. For example, the addition of appropriate booster sessions may strengthen or sustain the effects of the intervention and the use of mobile technology may facilitate self-report data collection and potentially enhance the evaluation of treatment fidelity, which is often missing in CAM mind-body intervention research. In addition, when examining measures of stress and associated symptoms in pregnant women, consideration should be given for the addition of pregnancy anxiety measures.

The results of this study also support the feasibility and acceptability of this intervention. Of those who met the inclusion criteria, 97% of women agreed to participate and, of those, 83% completed the 12-week study. The majority of participants (85%) reported at the beginning of the study that they were not using any stress management technique. Given the negative health outcomes associated with stress during pregnancy, it is imperative that effective, low-cost, and easy to use interventions be available to this population. GI, a CAM mind-body intervention, is a promising intervention to meet that need.

## Figures and Tables

**Table 1:** Demographic variables by group (*N* = 72).

Variable	GI *N* = 36 Mean (SE)	UC *N* = 36 Mean (SE)	Total *N* = 72 Mean (SE)	*P* value
Age	24.75 (1.06)	23.78 (0.73)	24.26 (0.64)	0.454
Height	5.31 (0.05)	5.38 (0.04)	5.35 (0.03)	0.315
Weight	176.56 (6.20)	171.49 (7.15)	174.03 (4.71)	0.594
Number of pregnancies	2.89 (0.31)	2.37 (0.30)	2.63 (0.22)	0.236
Weeks pregnant	15.53 (0.16)	15.32 (0.22)	15.43 (0.13)	0.446

	% (*N*)	% (*N*)	% (*N*)	

Income				
Less than 15,000	72% (26)	64% (23)	68% (49)	0.617
Between 15,000 and 44,999	25% (9)	28% (10)	26% (19)	
More than 45,000	3% (1)	8% (3)	5% (4)	
Marital status				
Married/married before	17% (6)	11% (4)	14% (10)	0.496
Single never married	83% (30)	89% (32)	86% (62)	
Employment				
Full-/part-time job	39% (14)	39% (14)	39% (28)	0.924
No Job	58% (21)	61% (22)	60% (43)	
Education				
High school or less	47% (17)	50% (18)	49% (35)	0.896
Posthigh school	36% (13)	39% (14)	37% (27)	
Postcollege	17% (6)	11% (4)	14% (10)	
Drink alcohol				
No	86% (31)	97% (35)	92% (66)	0.199
Yes	14% (5)	3% (1)	8% (6)	
Smoking				
No	75% (27)	89% (32)	82% (59)	0.220
Yes	25% (9)	11% (4)	18% (13)	
Beverages w/caffeine				
No	22% (8)	36% (13)	29% (21)	0.195
Yes	78% (28)	64% (23)	71% (51)	
Use stress management				
No	80% (28)	89% (32)	85% (60)	0.343
Yes	20% (7)	11% (4)	15% (11)	

**Table 2:** Least square means and standard errors from mixed linear model analysis comparing GI and UC groups.

Measurement	GI Mean (SE) *N* = 36	UC Mean (SE) *N* = 36	*P* values*
PSS			
Baseline	20.30 (1.41)	19.23 (1.36)	
Time 2	16.89 (1.42)	19.99 (1.37)	0.012
Time 3	17.11 (1.42)	17.48 (1.37)	0.381
STAI-S			
Baseline	39.59 (2.32)	39.35 (2.24)	
Time 2	36.42 (2.38)	39.10 (2.27)	0.382
Time 3	36.38 (2.35)	34.44 (2.30)	0.606
STAI-T			
Baseline	43.79 (2.32)	43.14 (2.26)	
Time 2	39.69 (2.36)	44.45 (2.26)	0.041
Time 3	39.11 (2.34)	39.35 (2.28)	0.735
BFI Total			
Baseline	5.26 (0.43)	4.55 (0.42)	
Time 2	4.04 (0.44)	4.59 (0.42)	0.040
Time 3	3.60 (0.44)	3.87 (0.43)	0.104

	Median (minimum and maximum) pg/mL *N* = 16	Median (minimum and maximum) pg/mL *N* = 15	

CRH			
Baseline	0.97 (0.03, 5.75)	0.85 (0.03, 9.49)	
Time 2	8.80 (1.06, 47.32)	8.30 (3.35, 85.29)	0.737^‡^
Time 3	36.47 (4.90, 87.17)	45.14 (2.78, 146.87)	0.827^‡^

*P values from mixed-effects linear model comparing the GI group change from baseline to 8 weeks and 12 weeks with the corresponding UC group change from baseline to 8 and 12 weeks.

^‡^ *P* values from mixed-effects linear model using the log CRH.

**Table 3:** Frequencies of GI CD tract usage from week 5 to week 12.

GI CD tract	Week 5	Week 6	Week 7	Week 8	Week 9	Week 10	Week 11	Week 12
1	55	64	38	38	59	50	38	42
2	42	45	55	45	44	46	44	39
3	44	40	45	52	34	39	46	34
4	30	34	35	42	38	34	26	53
% of participants reporting using CD during week	84.23%	90.15%	85.22%	87.19%	86.2%	83.25%	75.86%	82.75%
